# DNA mismatch repair system regulates the expression of PD-L1 through DNMTs in cervical cancer

**DOI:** 10.1186/s12935-024-03214-7

**Published:** 2024-01-10

**Authors:** Fan Guo, Ruijiao Lu, Weina Kong, Miyessar Anwar, Yangchun Feng

**Affiliations:** 1 Department of Medical Laboratory Center, Tumor Hospital Affiliated to Xinjiang Medical University, No 789 Suzhou Road, Urumqi, China; 2 Postdoctoral Research Workstation of Tumor Hospital affiliated to Xinjiang Medical University, Urumqi, China

**Keywords:** Cervical cancer, Programmed death-ligand 1, DNA mismatch repair

## Abstract

**Background:**

Cervical cancer (CC) is a potential clinical application of PD-1/PD-L1 inhibitor. We aimed to study the mechanism of DNA mismatch repair (MMR) system regulating the expression of PD-L1 in CC through DNA methyltransferase (DNMTs).

**Methods:**

We collected pathological specimens from 118 cases of CC to analyze the relationship between PD-L1 expression and DNMTs in different MMR states. RNA interference (RNAi) technique was used to simulate the formation of CC cell line with MMR deficiency (dMMR) state, and subcutaneous tumor formation experiment was carried out in nude mice to verify the relationship between PD-L1 expression and DNMTs in MMR state.

**Results:**

The PD-L1 positive rate in 118 cases of CC was 58.47%, while the microsatellite instability (MSI) status accounted for 5.93%. There was a significant difference in the expression of PD-L1 between patients within the dMMR and MMR proficient (pMMR) groups (χ^2^ = 21.405, *P* < 0.001). Subcutaneous inoculation after infection of Siha cells led to successful tumorigenesis in nude mice, accompanied by a significant increase in the level of PD-L1 expression in the mouse tumors, while the expression level of MLH1 and MSH2 protein decreased significantly. We also found that PD-L1 expression was closely related to the expression of DNMTs.

**Conclusion:**

PD-L1 is universal expressed on the surface of CC cells, dMMR status enhances the expression of PD-L1 on the surface of CC cells, dMMR states of CC are related to the demethylation status of the PD-L1 gene promoter region.

## Introduction

Cervical cancer is one of the most common cancers in the female reproductive system, originating from the cervix. It is a serious threat to women’s health and is the most prevalent tumor in 23 countries [1]. CC has an incidence rate of 604,127 new cases and 341,831 deaths in 2020 as reported by the GLOBOCAN study. The incidence and mortality rates of CC in China are on the rise [2], and the age of onset is gradually decreasing. Furthermore, Xinjiang is a high-incidence area for CC, so finding molecular markers that can be used in the diagnosis and treatment of CC has become a priority research.

Study shows MMR proteins exist in CC, and dMMR genes may be one of the important mechanisms of cervical carcinogenesis [3]. MMR is a highly conservative biological pathway, MMR corrects DNA mismatches and promotes DNA damage response signaling during DNA replication, which plays a key role in maintaining genomic stability [4]. MMR has been widely reported in endometrioid, ovarian, colorectal, and pancreatic cancers [5–7]. dMMR has also been found to impact the prognosis of ovarian and colorectal cancers. Baretti et al., found that patients with high microsatellite instable (MSI-H) and microsatellite stable (MSS) tumors had a better prognosis, especially in stage II and III disease [8, 9]. dMMR often leads to MSI, which is an important predictive biomarker for the therapeutic efficacy of cancer immunotherapy. Recent studies have found that PD-1/PD-L1 inhibitors, an immune checkpoint inhibitor, can be used in solid tumors with dMMR such as colorectal cancer [10], but its potential application in CC is worth further investigation. The relationship between dMMR and clinical prognosis of CC is unclear. This study systematically evaluates the current status of research on the MMR gene as a prognostic factor in CC, aiming to comprehensive understand the impact of dMMR on the prognosis of CC and provide reference for subsequent CC prognosis and medication.

## Materials and methods

### Patients

In this study, 118 cases who underwent CC surgery in the Third Clinical Medical College of Xinjiang Medical University (Affiliated Tumor Hospital) from January 2012 to December 2014 were retrospectively collected, paraffin pathology specimens and frozen pathology specimens of the patients were taken as the subjects of the study. The clinical data of the patients were also compiled, including age, ethnicity, pregnancy history, reproductive history, differentiation degree of tumor cells (TCs), size and FIGO stage. The experiment was approved by the Ethics Committee of the Third Clinical Medical College of Xinjiang Medical University (Affiliated Tumor Hospital). (1) Inclusion criteria: patients who underwent CC surgery in the Third Clinical Medical College of Xinjiang Medical University with complete data. (2) Exclusion criteria: patients with a history of chemotherapy or radiotherapy; patients who had received cervical physiotherapy; patients with autoimmune diseases.

### Immunohistochemistry (IHC)

The paraffin sections were deparaffinized, hydrated, and microwave-repaired. Indicated primary antibodies (rabbit anti-human PD-L1, 1:100, ab205921, abcam; rabbit anti-human MLH1, 1:100, ab92312, abcam; rabbit anti-human MSH2, 1:800, ab70270, abcam; rabbit anti-human MSH3, 1:200 bs-4919R, Beijing Boosun Biotechnology Co., Ltd; rabbit anti-human MSH6, 1:500, ab92471, abcam) and biotin-labeled secondary antibodies (PV-6001 kit, Beijing Zhongsugjinqiao Biotechnology Co., Ltd.) were serially added, incubation at 7 °C for 45 min, addition of horseradish enzyme-labeled streptavidin working solution (incubation for 15 min). Then, diaminobenzidine (DAB) development (Beijing Zhongsui Jinqiao Biotechnology Co., Ltd.), hematoxylin counterstaining, further dehydration, and transparency, all the operations were carried out in accordance with the instructions of the kit. The staining results were independently judged by two pathologists, in order to confirm their reproducibility, 25% of sections were randomly selected and scored twice. The percentage of PD-L1 positivity was calculated by counting 100 TCs at high magnification mirror, with less than 10% being negative, more than 10% being positive, and more than 50% being classified as strongly positive for PD-L1 in TCs. MMR protein was counted by taking ten fields of view each of the tumor tissue aggregates at high magnification mirror, and samples with less than 1% positive cells in the field of view were scored as MLH1, MSH2, MSH3, or MSH6 negative, whereas samples with more than 1% positive cells were scored as MLH1, MSH2, MSH3, or MSH6 positive.

### Microsatellite instability (MSI)

The length polymorphism analysis of fluorescent labeled amplification products was used for MSI detection. A total of 9 pairs of PCR primers were designed to form 2 PANELs and to amplify of 9 polymorphic loci. Fluorescent labeling of the loci was performed by 5’FAM fluorescence, including a total of 8 STR loci and 1 sex locus typing. The primers for this study were designed using the online Primer3 software (design scheme: http://frodo.wi.mit.edu/cgi-bin/primer3/ primer3_www.cgi) and synthesized at Shanghai Tianhao Biotechnology Co. A small amount of PCR product was taken and mixed with the internal standard marker, ABI3730xl was used for capillary electrophoresis, and the final data file was analyzed by GeneMapper5.0 (Applied Biosystems).

### Gene mutation analysis in TCGA database

The cBio Cancer Genomics Portal (cBioPortal) database was used to identify possible MMR copy number variations and mutations in CC. At the same time, the relationship between these mutations and the prognosis of CC patients was analyzed.

### Quantitative real-time PCR (qRT-PCR)

According to the manufacturer’s instructions, total RNA was extracted and cDNA was reverse-transcribed. The PCR reaction system was performed using SYBR Premix Ex Taq (1 µl DNA template + 1x HotStarTaq buffer, 0.1 µM primer mix, 3.0 mM Mg^2+^, 0.3 mM dNTP, 1 U HotStarTaq polymerase) (Qiagen Inc.). Quantitative PCR was performed in a LightCycler 480 instrument (Roche, Switzerland). The PCR process consisted of a three-step procedure, starting with a preheating at 95ºC for 2 min, followed by 11 cycles with denaturation at 94ºC for 20 s, annealing at 62ºC for 4 s, and extension at 68ºC for 2 min. Then, followed by 24 cycles with denaturation at 94ºC for 2 s, annealing at 56ºC for 3 s, and extension at 68ºC for 2 min. GAPDH was used as an internal reference, and the 2-ΔΔCT method was used to calculate the relative expression of PD-L1 and DNA methyltransferase mRNA. Primer sequences are listed in Table 1.

**Table 1:** Primer sequence

Gene	Primer sequence	
GAPDH	Forward	5’-TGACTTCAACAGCGACACCCA-3’
	Reverse	5’-CACCCTGTTGCTGTAGCCAAA-3’
PD-L1	Forward	5’-ACTGGCATTTGCTGAACG-3’
	Reverse	5’-TCCTCCATTTCCCAATAGAC-3’
DNMT1	Forward	5’-CCTAGCCCCAGGATTACAAGG-3’
	Reverse	5’-ACTCATCCGATTTGGCTCTTTC-3’
DNMT3a	Forward	5’-TCGCTAATAACCACGACCAG-3’
	Reverse	5’-ACACCTCCGAGGCAATGTAG-3’
DNMT3b	Forward	5’-CCCCTCCCAGCTCTTACCTTA-3’
	Reverse	5’-TCTCCACTGTCTGCCTCCACC-3’

### Western blot (WB)

Cell lines were washed with PBS, lysed in RIPA lysis buffer (WB-0071, Shanghai Dingguo Biotechnology Co., Ltd), the protein quantification was determined using a BCA Protein Concentration Measurement Kit (Biyuntian) according to the manufacturer’s instructions. RIPA lysis buffer was diluted to adjust the concentration of sample proteins, and loading buffer was added to make the final concentration of 2 µg/µL, polyacrylamide gel electrophoresis (PAGE) (Shanghai Tianneng Company) 400 mA electrotransfer 2 h (the loading amount of DNMTs protein antigen up-sampling in CC patients was 40 ug, and the loading amount of DNMTs and PD-L1 protein antigen up-sampling in dMMR CC cells was 50 ug). Primary antibody incubation was conducted at 4 ℃ overnight in blocking solution (Dilution ratio: 1:1000), TBST wash the membrane, the secondary antibodies in working solution contained biotin-labeled goat anti-mouse IgG (1:10000, Shanghai Genechem Co., Ltd, China) or biotin-labeled goat anti‐rabbit IgG (1:10000, Shanghai Genechem Co., Ltd, China), 20X LumiGLO ® Reagent and 20X Peroxide #7003 (CST public) for development.

### Cell culture and lentivirus transfection

Siha and Caski CC cells were recovered by conventional methods and cultured in DMEM medium (containing 10% FBS and 1% penicillin-streptomycin) at 37 °C and 5% CO2 until the cell confluence reached about 80% and then cell particles were collected. The target gene RNA interference lentivirus and negative control lentivirus were designed and constructed by Shanghai Jinhua Co., Ltd. The target sequence is shown in Table 2. Siha and Caski CC cell lines (adherent or suspension cell line) in the logarithmic growth phase were digested with 0.25% trypsin, and continued to be cultured after inoculation respectively until the electroplating volume reached 15–30% to ensure infection. The experiment was divided into 3 groups: including MLH1-KD, MSH2-KD, and NC groups. Added the optimal amount of virus for infection. After 72 h of transfection, cell state and transfection efficiency were visualized under a fluorescence microscope.

**Table 2:** RNAi sequence

Serial number	Target sequence
MLH1-RNAi(90137-1)	gtGTTCTTCTTTCTCTGTATT
MLH1-RNAi(90138-2)	CTGGAAGTGGACTGTGGAACA
MLH1-RNAi(90139-1)	CGGGAAGATTCTGATGTGGAA
MSH2-RNAi(81799-1)	gcCTTGCTGAATAAGTGTAAA
MSH2-RNAi(81800-1)	ccTGGCAATCTCTCTCAGTTT
MSH2-RNAi(81801-1)	gcATGTAATAGAGTGTGCTAA

### Nude mice develop tumors

Each mouse was inoculated with 200 uL of TCs suspension subcutaneous injection (2.5 × 10 ^7^ cells/ml). BALB/c nude mice (4 weeks old, Shanghai Genechem Co., Ltd, China) were used to establish the subcutaneous transplantation model. After the tumors were formed, vernier calipers were used to measure and record the long and short diameters of the tumors in each group of nude mice. The tumor volume (volume = 0.5 × long diameter × short diameter × short diameter) was calculated, and the growth curve was drawn by taking the average value of each group of tumors. The nude mice were killed by spinal dissection after 37 days, and the tumors were completely peeled off and weighed.

### Statistical analysis

GraphPad Prism 8.0 software and SPSS 26.0 software were used for data statistical analysis. Measurement data that conform to a normal distribution are represented by mean ± standard deviation and used to create a bar chart. One-way analysis of variance was used to compare the mean among groups of data which conform to normal distribution, and Tukey test was used for pairwise comparison between two groups of data. Pearson Chi-square test or Fisher exact test were used for comparison between groups with counting data. Linear correlation or Spearman correlation analysis was used for correlation between two variables. Kaplan-Meier method was used to draw the survival curve, and Log-rank test was used to evaluate the survival difference between the two groups. Tumor volume was analyzed by Two-way repeated measures ANOVA, and tumor weight was analyzed by Welch one-way ANOVA. A two-tailed test was used for all analyses. Statistical significance was indicated at *P* < 0.05.

## Results

### Expression of PD-L1 in pathological specimens of cervical cancer

**Fig. 1:**
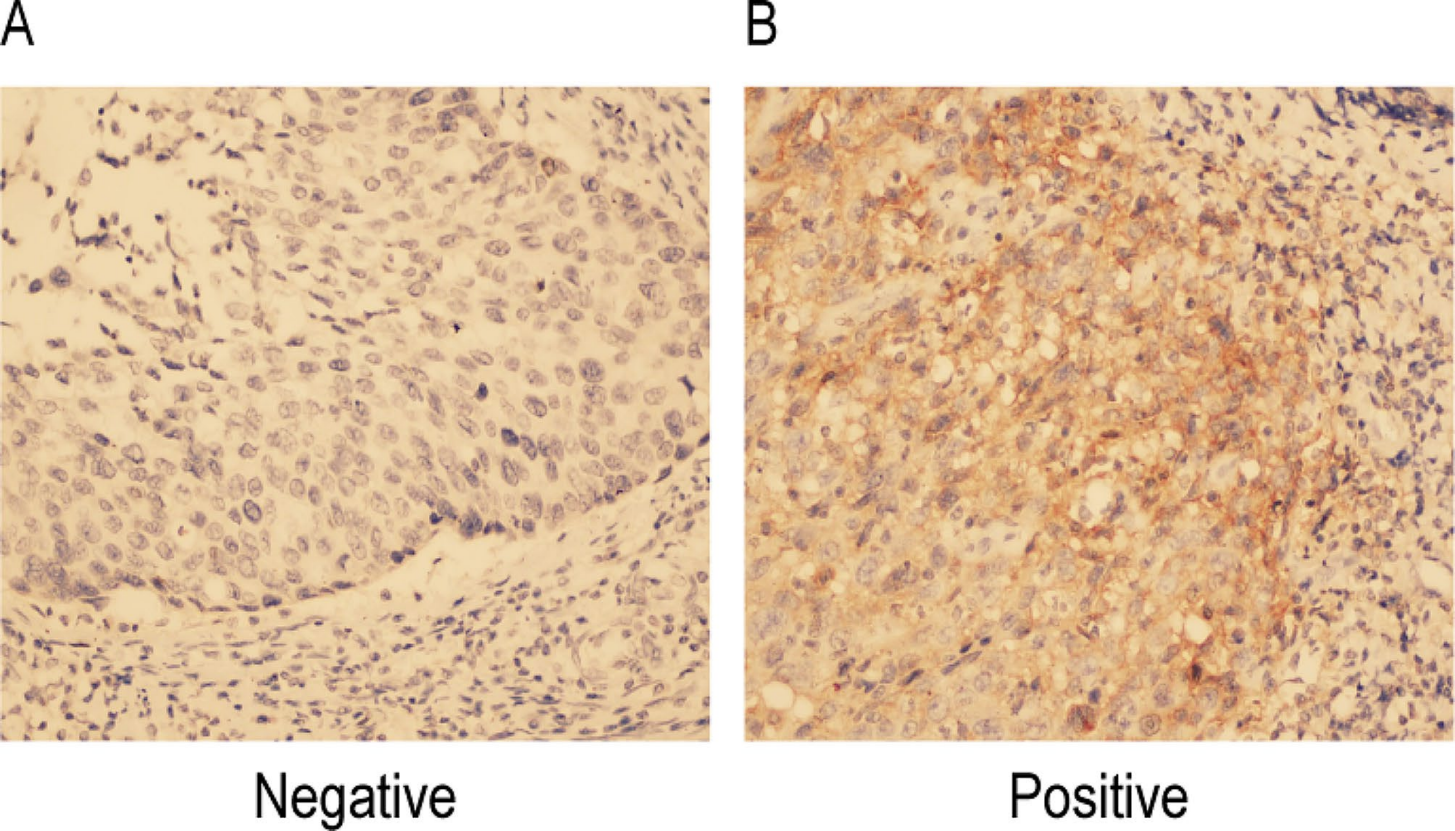
PD-L1 in cervical cancer cell result of IHC results.(× 400) **(A)** PD-L1 negative expression. **(B)** PD-L1 positive expression

The expression of PD-L1 protein in CC tissues was mainly located in the cell membrane and cytoplasm, DAB showed brown color after color development (Fig. 1B). Among the 118 CC samples studied, 49 cases (41.53%) were PD-L1 negative. 49 cases (41.53%) were positive. 20 cases (16.94%) were strongly positive. Positive and strong positive accounted for 58.47% (Table 3). The expression level of PD-L1 in TCs was significantly different between dMMR and pMMR groups (χ^2^ = 21.405, *P* < 0.05), suggesting that the change of MMR status was closely related to the expression of PD-L1 protein in TCs of CC.

**Table 3:** Compared to PD-L1 expression of tumor cells in different MMR groups

MMR group	Number of cases	PD-L1 expression*		
		Negative	Positive	Strong positive
dMMR	34	5	16	13
pMMR	84	44	33	7
total	118	49	49	20

*χ^2^ = 21.405, *P* < 0.05

### Frequency of MSI

**Fig. 2:**
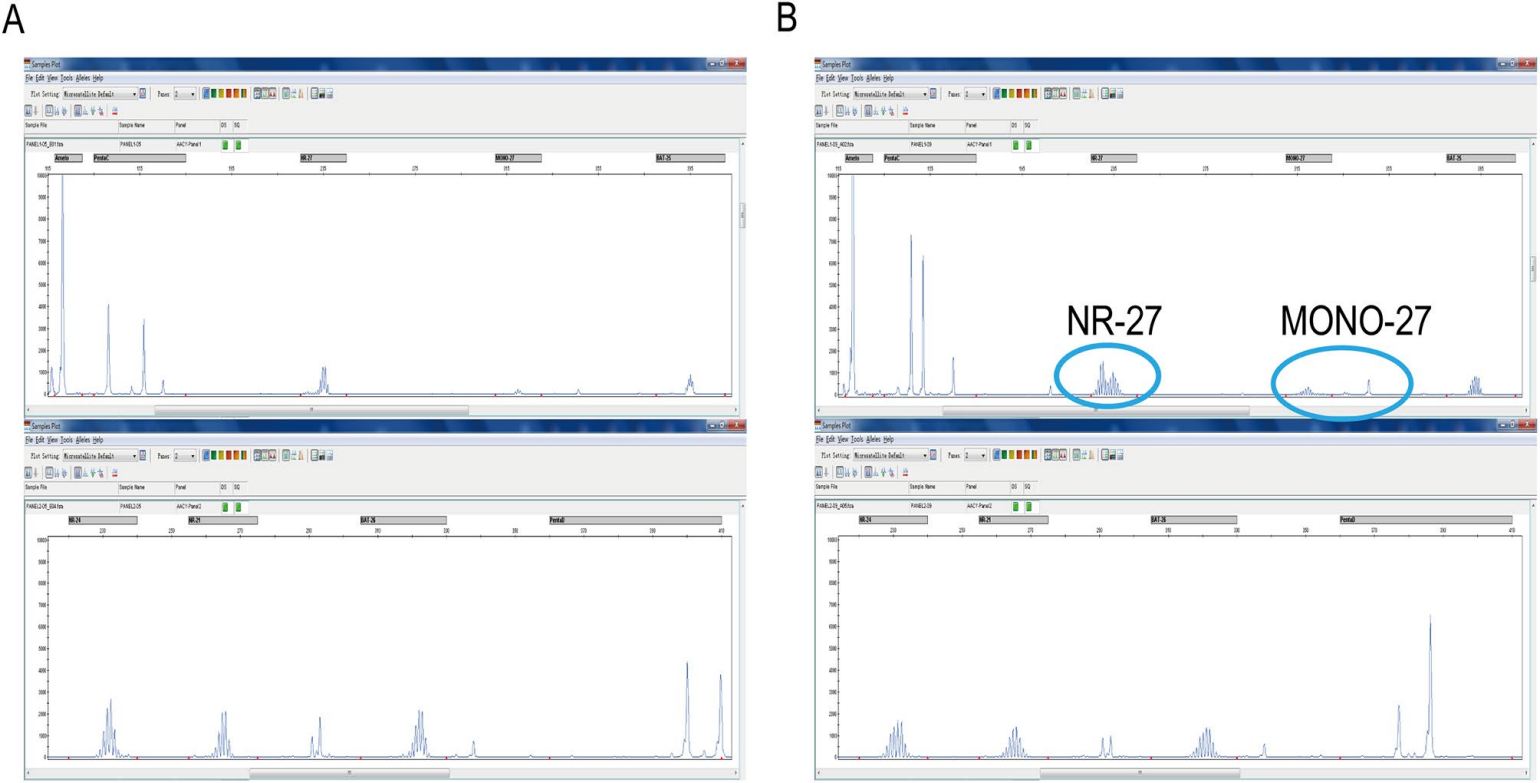
MSI test of cervical cancer. (**A)** Microsatellite stable (MSS). (**B)** Microsatellite instability (MSI).

The assessment of MSI status is shown in Fig. 2. A total of 7 cases of MSI status were present in 118 specimens, with an incidence of 5.93%, at the MONO-27 and NR-27 loci.

### Expression of MMR protein in cervical cancer

**Fig. 3:**
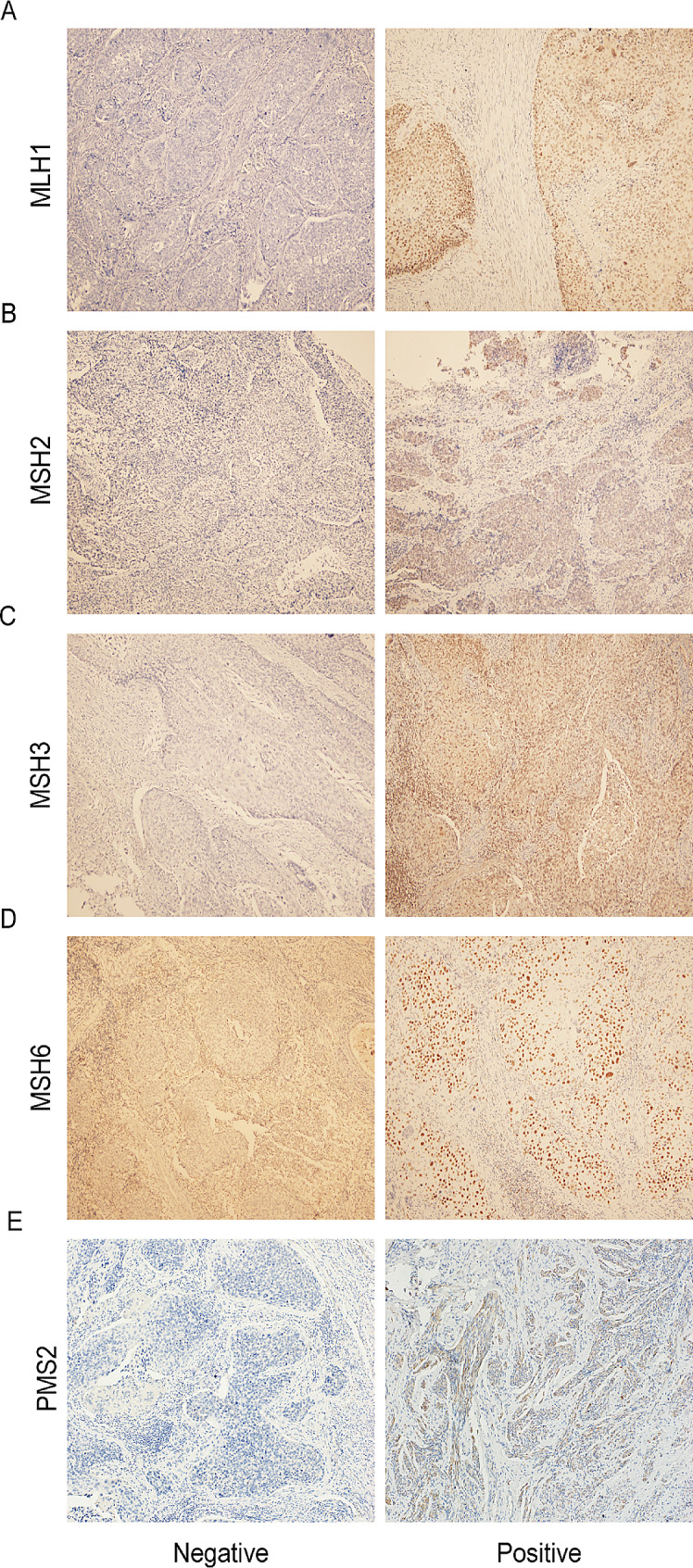
Expression of DNA mismatch repair protein in pathological specimens (× 100). **(A)** MLH1 result of IHC results. **(B)** MSH2 result of IHC results. **(C)** MSH3 result of IHC results. **(D)** MSH6 result of IHC results. **(E)** PMS2 result of IHC results

MMR protein was mainly expressed in the nucleus and was represented by a yellowish-brown color after staining (Fig. 3). Among the 118 samples, there were 34 cases (28.81%) of dMMR and 84 cases (71.79%) of pMMR. Among them, 84 (71.2%) specimens were positive for MLH1, MSH2, MSH3, MSH6, and PMS2; 29 (24.58%) specimens showed a single deletion of MLH1, MSH2, MSH3, MSH6, or PMS2; 4 (3.37%) specimens showed a deletion of both MLH1 and MSH2 expression; 1 (0.85%) specimen showed a deletion of MSH2 and MSH3 co-expression.

### MMR gene mutation in TCGA database

**Fig. 4:**
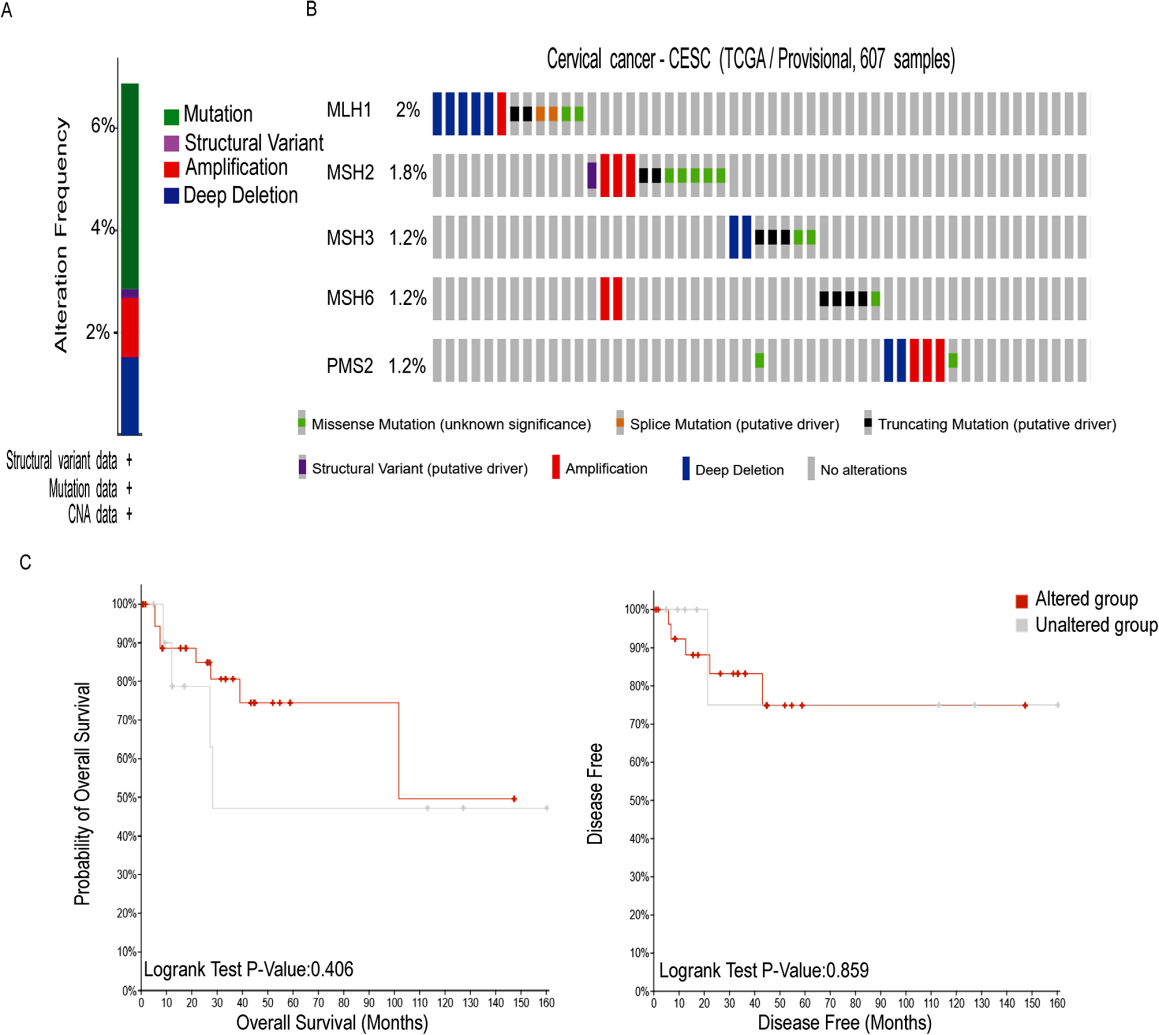
MMR gene mutations in cervical cancer analyzed by the cBioPortal database. **(A)** Frequency of MMR gene mutations in cervical cancer. **(B)** Mutation rates of MLH1, MSH2, MSH3, MSH6 and PMS2. **(C)** Correlation between genetic mutations and patients’ overall survival (OS) and Disease Free Survival (DSS).

MMR gene alterations were found in 41 (6.89%) of 595 CC patients in the TCGA database (Fig. 4A). Among them, the mutation rate was 2% for MLH1, 1.8% for MSH2, 1.2% for MSH3, 1.2% for MSH6, and 1.2% for PMS2. Through research on mutations and patient survival prognosis, we have not found that these genetic mutations were related to patient prognosis and survival. Because the sample size of mutations corresponding to gene expression profiles in the TCGA database was not large enough, which may limit the reliability of the results.

### Relationship between the expression of MMR protein and PD-L1 and clinicopathological features in cervical cancer

From Table 4, the presence of a history of miscarriage and births ≥ 3 in CC patients was associated with the dMMR status of TCs (χ^2^ = 4.458, *P* = 0.035; χ^2^ = 11.356, *P* = 0. 001), and the results indicated that the presence of a history of miscarriage and births ≥ 3 in CC patients was associated with a higher incidence of dMMR status of TCs. However, PD-L1 expression status in CC was closely associated with the presence of a history of miscarriage and low differentiation status (Z = -4.404, *P* = 0.000; Z = -3.644, *P* = 0.000).

**Table 4:** Relationship between MMR/PD-L1 and clinicopathological features of cervical cancer

Variable	Total cases	MMR		*P*	PD-L1			*P*
		dMMR	pMMR		Strongly positive	Positive	Negative	
CESC	118	34	84		20	49	49	
Age (years)				0.041				0.451
<35	20	3	17		0	11	9	
35–55	78	21	57		15	33	30	
>55	20	10	10		5	5	10	
Childbearing history (times)				0.001				0.279
0–2	60	9	51		7	21	32	
≥ 3	58	25	33		13	28	17	
Abortion history				0.035				0.000
Yes	69	25	44		18	31	20	
No	49	9	40		2	18	29	
Differentiation				0.994				0.000
Low	66	19	47		11	31	24	
Middle/High	52	15	37		9	18	25	

### Comparison of DNA methyltransferase relative expression levels in patients with cervical cancer

**Fig. 5:**
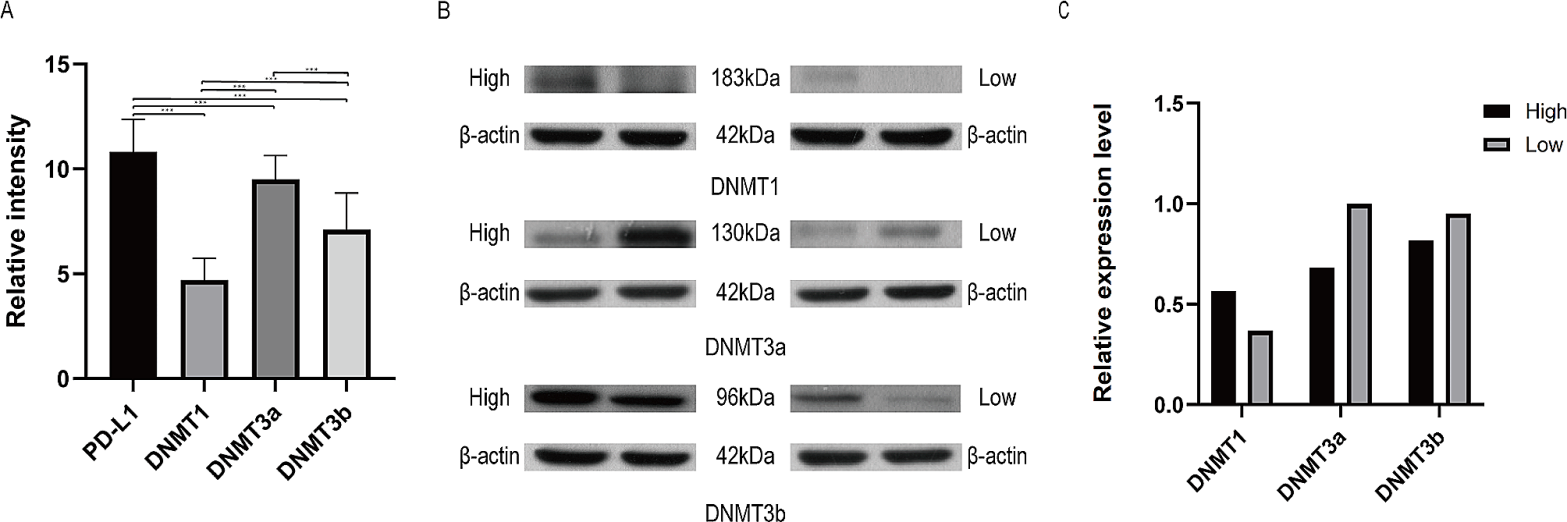
Correlation analysis between PD-L1 expression and DNA methyltransferase. **(A)** Expression of PD-L1 and DNMTs genes in cervical cancer tissues. **(B)** Expression analysis of DNMTs protein. **(C)** Quantitative results of the relative expression of DNMTs protein detected by western blot (ns: *P* > 0.05, *P ≤ 0.05, **P ≤ 0.01, ***P ≤ 0.001)

The relative expressions of DNMT1, DNMT3a, and DNMT3b in CC patients were 4.71, 9.51, and 7.11. The expression of PD-L1 was 10.82 (Fig. 5A). Comparison of the correlation analysis of PD-L1 expression profile with DNMTs showed that the expression of PD-L1 in TCs was closely correlated with the expression of DNMTs (DNMT1, DNMT3a, DNMT3b) (*P* < 0.0001). WB assay results showed that DNMT3a protein expression had the most significant difference between the high and low expression groups, as PD-L1 protein expression increased, DNMT1 and DNMT3a protein expression also increased, whereas DNMT3b expression decreased (Table 5).

**Table 5:** Comparison of correlation between PD-L1 expression and DNMTs

DNMTs	PD-L1	
	*r*	*P*
DNMT1	0.43	<0.0001
DNMT3a	0.51	<0.0001
DNMT3b	-0.63	<0.0001

### Transfection efficiency of Siha and CaSki cells in cervical cancer by RNAi technology

The mRNA assay to evaluate the inhibition of gene expression by RNAi is shown in Table 6. We found that in Siha cells, compared with the NC group, the MLH1 gene knockdown efficiency in the KD1 group reached 69.6%, and the MSH2 gene knockdown efficiency reached 85.5%. In Caski cells, compared with the NC group, the MLH1 gene knockdown efficiency in the KD1 group reached 76%, and the MSH2 gene knockdown efficiency reached 83.7%. After post-transfection screening, we found that MLH1 mRNA expression was only 24% and 30.4% of normal Caski and Siha cells, and for MSH2 mRNA, its mRNA expression was only 16.2% and 14.1% of normal Caski and Siha cells, indicating that the effect of shRNA inhibition of MLH1 and MSH2 was better, it can be used as the basis for subsequent experimental research on gene knockdown of CC cells.

**Table 6:** mRNA assays assess RNA interference with inhibition of gene expression

Group	Siha			Caski	
	MLH1-mRNA	MSH2-mRNA		MLH1-mRNA	MSH2-mRNA
NC	1.001	1.026		1.001	1.008
KD1	0.304	0.145		0.240	0.163
KD2	0.506	0.449		0.460	0.246
KD3	0.643	0.965		0.463	0.443

**Fig. 6:**
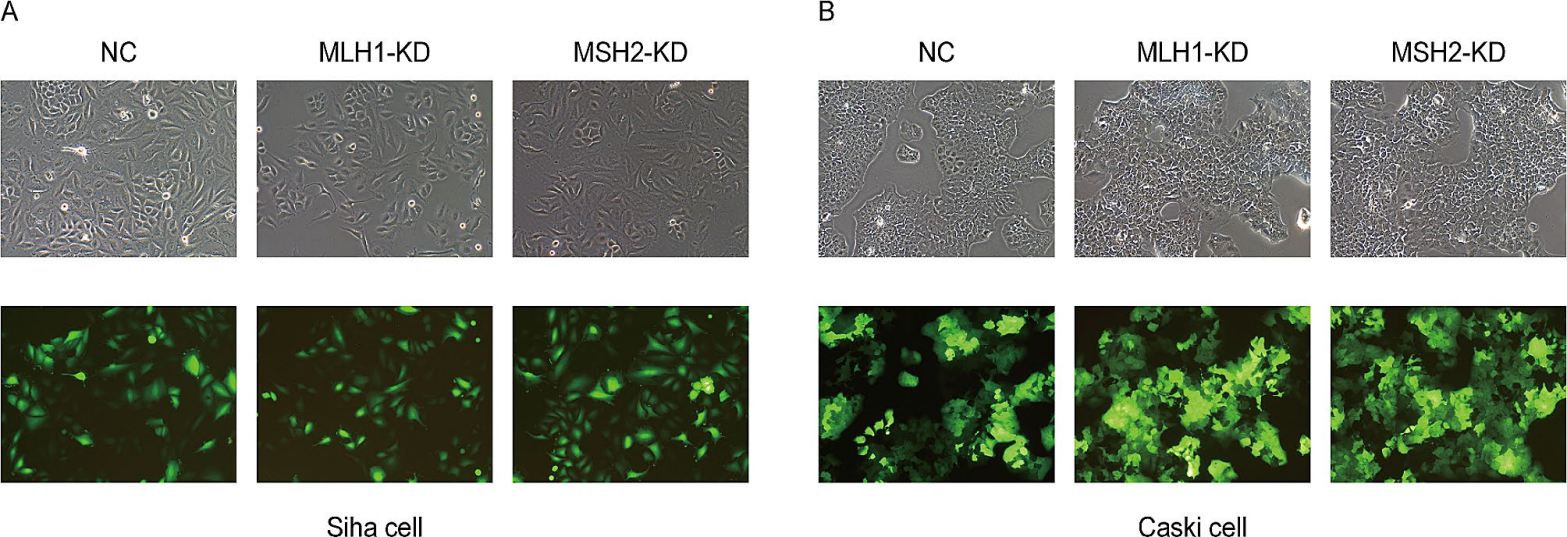
Bright field and fluorescence field images of Siha and Caski cells infected with lentivirus (× 100). (**A)** Transfection of lentiviral particles in NC, MLH1-KD and MSH2-KD groups of Siha cells (bright field and fluorescence field observation map). (**B)** Transfection of lentiviral particles in NC, MLH1-KD and MSH2-KD groups of Caski cells (bright field and fluorescence field observation map) (Green fluorescent protein (GFP) was excited at 488 nm, and the emitted light was captured at 507 nm)

Then, Siha and Caski cells were infected with NC, MLH1-KD, and MSH2-KD knockdown lentiviruses respectively. Figure 6 showed the bright field and fluorescence field observation of lentiviral infection. By observing the number of cells expressing fluorescence, the transfection efficiency of cells was obtained. We found that the transfection efficiency of lentivirus in Siha cell was 85% and 83%, and that in Caski cell was 83% and 87%. Respectively, which met the needs of the RNAi.

### Expression of DNMTs and PD-L1 in dMMR CC cells

**Fig. 7:**
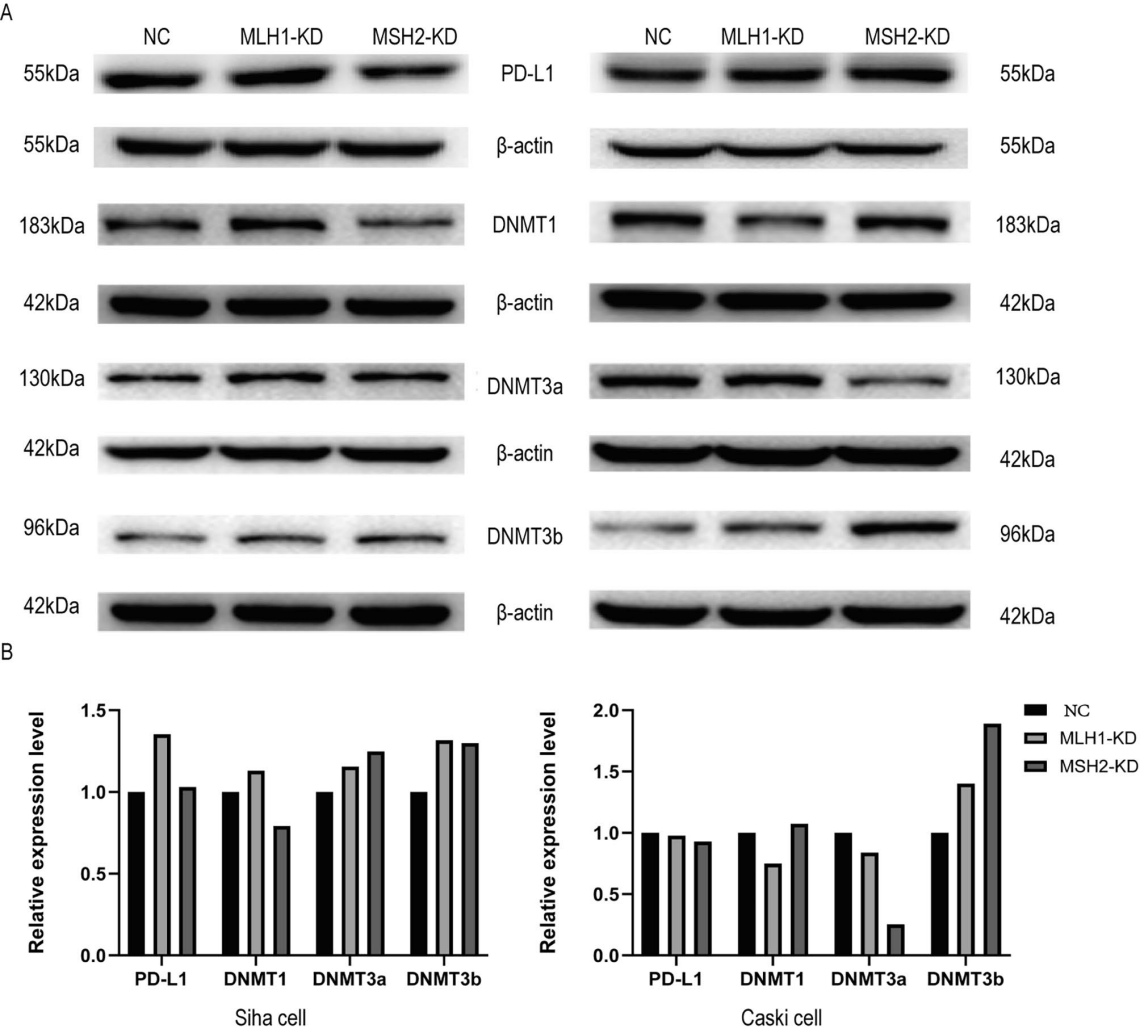
Expression analysis of PD-L1 and DNMTs protein in Siha and Caski cells. **(A)** Detection of PD-L1 and DNMTs expression in Siha and Caski by Western blot. **(B)** Quantitative results of the relative expression of PD-L1 and DNMTs protein in Siha and Caski detected by western blot.(All the results here are relative quantitative results corrected by NC group)

The mRNA expression of DNMTs and PD-L1 in dMMR CC cells were shown in Tables 7 and 8, combined with the protein expression results of WB (Fig. 7), we found that the mRNA expression of PD-L1 and the level of PD-L1 protein expression in Siha cells were different. However, there was no statistically significant difference among the three groups in Caski cells. We also found that DNMTs expression was closely related to PD-L1 expression, suggesting that the dMMR system was associated with the demethylation status of the promoter region of the PD-L1 gene.

**Table 7:** Expression of DNMTs and PD-L1 mRNA in Siha cervical cancer cells under dMMR status

Group	DNMTs expression				PD-L1 expression
	DNMT1	DNMT3a	DNMT3b		PD-L1
NC	1.014	1.029	1.007		1.012
MLH1-KD	1.895	2.685	3,037		1.991
MSH2-KD	1.74	2.284	2.703		1.688

**Table 8:** Expression of DNMTs and PD-L1 mRNA in Caski cervical cancer cells under dMMR status

Group	DNMTs expression				PD-L1 expression
	DNMT1	DNMT3a	DNMT3b		PD-L1
NC	1.005	1.008	1.001		1.006
MLH1-KD	1.493	0.44	1.237		0.084
MSH2-KD	0.79	0.689	0.681		1.477

### Tumor growth of subcutaneous transplantation in nude mice

**Fig. 8:**
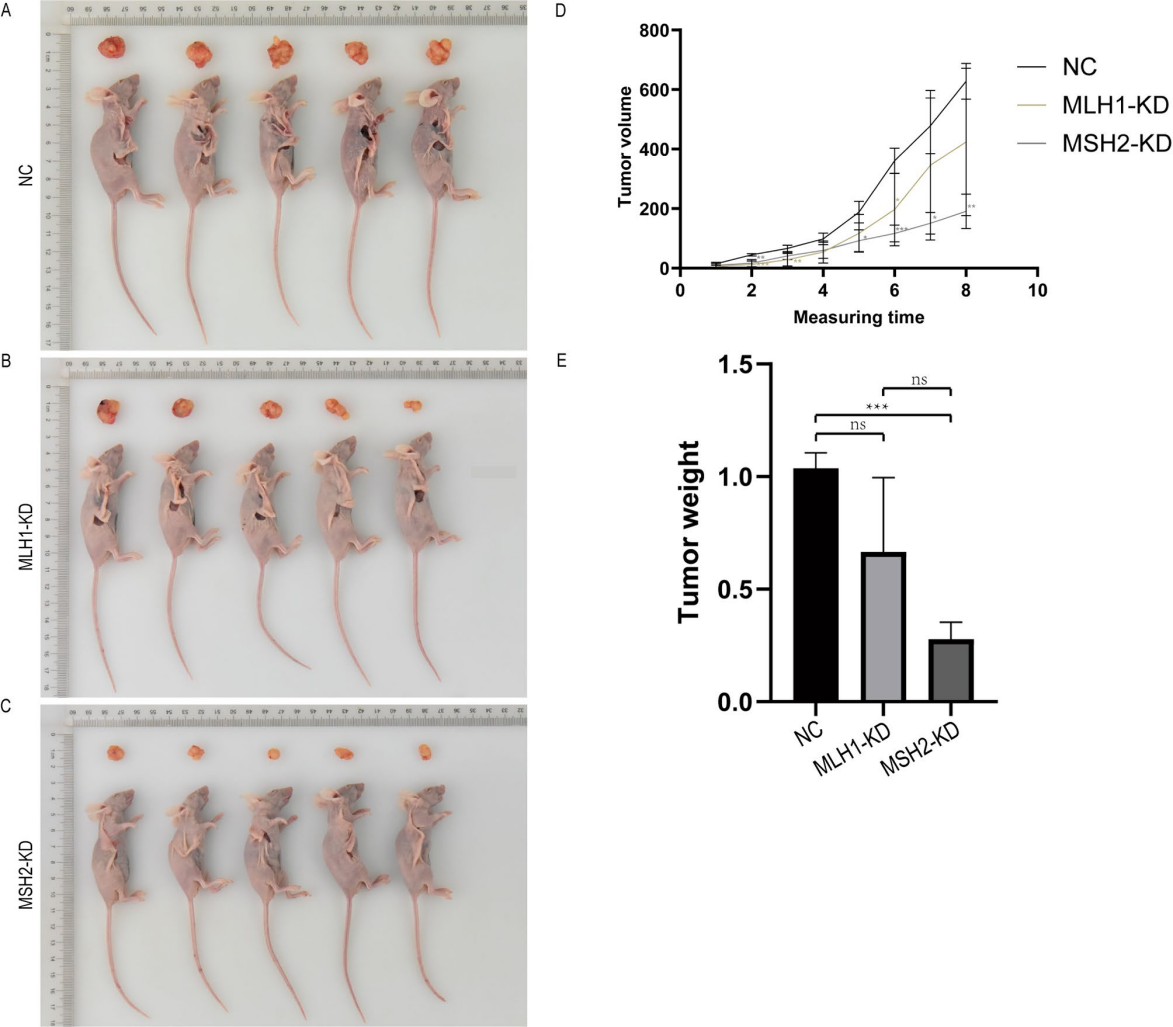
Analysis of tumorigenesis in nude mice in Siha cell group. **(A)** Tumorigenesis of Siha cells (NC + MLH1-KD + MSH2-KD group) in nude mice. **(B)** The tumor growth curve of nude mice. **(C)** Tumor weight of three groups (ns: *P* > 0.05, *P ≤ 0.05, **P ≤ 0.01, ***P ≤ 0.001)

As shown in Fig. 8A, Siha cells were more effective in inducing tumor formation in nude mice by subcutaneous inoculation after viral infection, when compared with the NC group, the tumor weight was no change in the MLH1-KD group (*P*>0. 05), while the tumor weight was decreased in the MSH2-KD group (*P* < 0.05); compared to the MLH1-KD group, there was no change in tumor weight in the MSH2-KD group (*P* > 0.05). The tumor growth curves over time were shown in Fig. 8B, and Fig. 8C showed the weight of the tumors in the three groups. The Caski cells were not tumorigenic in nude mice after subcutaneous inoculation by NC virus infection, and therefore were not included in the subsequent experiments.

### Expression of PD-L1, MLH1 and MSH2 in transplanted tumors after Siha cell-lentivirus transfection

**Fig. 9:**
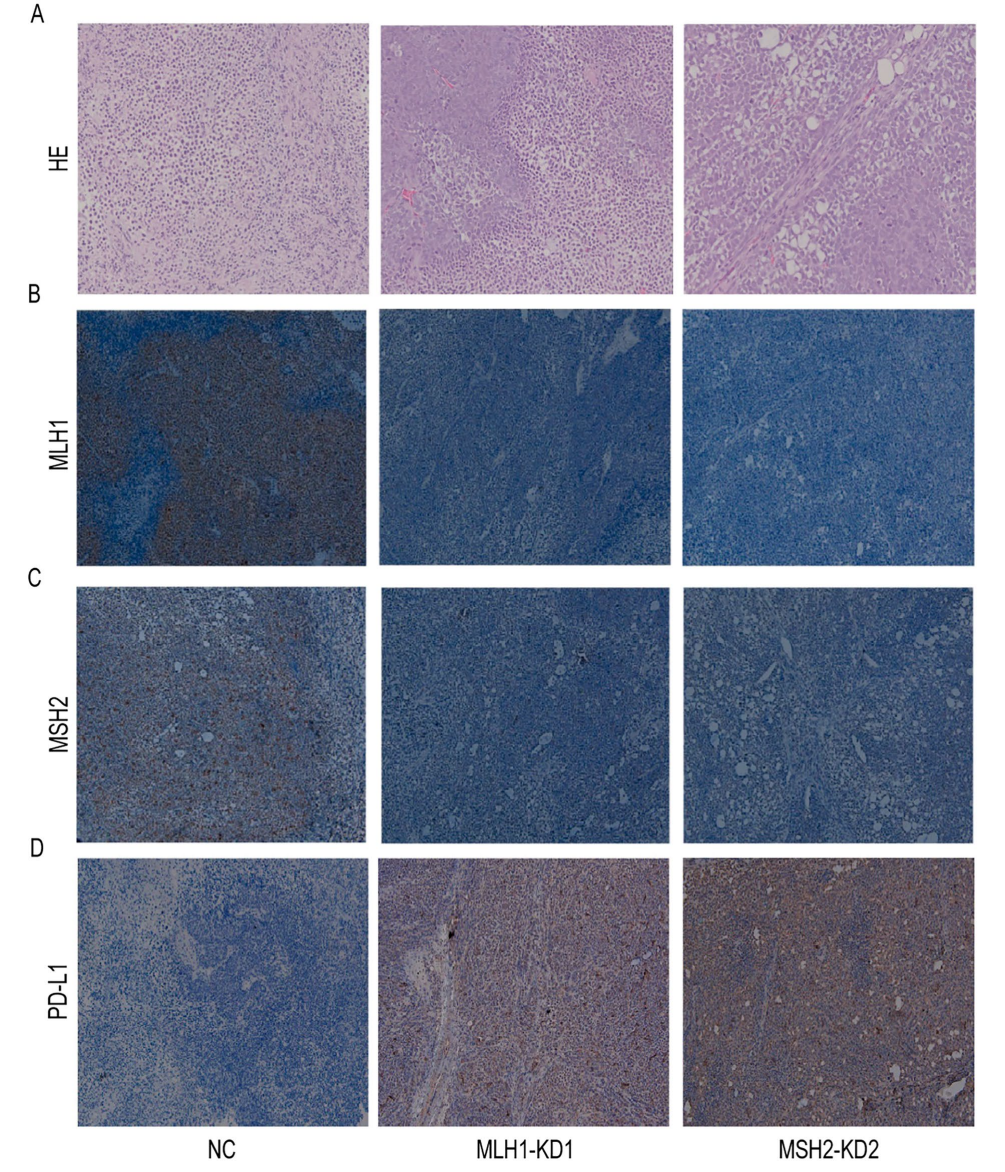
Staining results of pathological specimens of nude mouse tumors in Siha cell groups. (× 100) **(A)** HE staining results of NC, MLH1-KD and MSH2-KD groups. **(B)** MLH1 staining results of NC, MLH1-KD and MSH2-KD groups. **(C)** MSH2 staining results of NC, MLH1-KD and MSH2-KD groups. **(D)** PD-L1 staining results of NC, MLH1-KD and MSH2-KD groups

As shown in Fig. 9B and C, MLH1 and MSH2 protein expression in mouse tumors were located in the nucleus. The results of the present study showed that 5 samples in the NC group were positive for MLH1 and MSH2, 5 samples in the KD1 group were negative for MLH1 and 3 samples were negative for MSH2, and 4 out of 5 samples in the KD2 group were negative for MSH2 and 2 samples were negative for MLH1, with dMMR of 80%. PD-L1 expression was highly positive in both the KD1 and KD2 groups, with a positive rate of 100%.

Quantitative PCR revealed that the MLH1 gene expression level in the Siha cells MLH1-KD group was 0.495 times higher than that in the NC group, and the MSH2 gene expression level in the Siha cells MSH2-KD group was 0.495 times higher than that in the NC group. The level of PD-L1 gene expression in the Siha cells MLH1-KD group was 1.348 times higher than that of the NC group, and the level of PD-L1 gene expression in the Siha cells MSH2-KD group was 1.263 times higher than that of the NC group (Table 9).

**Table 9:** The expression of MLH1, MSH2 and PD-L1 in mouse tumors was detected by PCR

Group	MLH1	MSH2	PD-L1
NC	1.007	1.007	1.005
MLH1-KD	0.495	0.352	1.348
MSH2-KD	——	——	1.263

## Discussion

MMR system was first discovered in bacteria and yeast, then found in higher eukaryotes, its role is mainly to repair base mismatches occurring in the process of DNA replication, to enhance the high fidelity of DNA replication, so as to achieve the purpose of maintaining the stability of the genome [11]. The major proteins of the MMR system include MLH1, MSH2, MSH6, and PMS2 [12]. MLH1 is located at 3p21.3–23, which plays an important role in the process of mitosis and meiosis, is mainly involved in mismatch repair during mitosis, and its mutation is associated with Lynch syndrome. In a study conducted in South American countries, among the 220 screened mutations, MLH1 accounted for 54%. MLH1 and MSH2 mutations in the Chinese population accounted for 90% of Lynch syndrome mutations [13]. MLH1 and MLH3 play a crucial role in the segregation of homologous chromosomes during meiosis by ensuring cross-specific resolution of double Holliday junctions [14]. As the earliest discovered and most studied gene, MSH2 is also the most abundantly expressed gene among these proteins, which has the function of recognizing mismatches and the presence of MSH2 protein helps to stabilize MSH6 and MSH3 [15]. MSH6, also known as G/T binding protein or p160, completes mismatch repair by recognizing mismatches, assembling restorations, degrading mismatched DNA strands and repairing synthesis. It has been reported that MSH6 mutations could increase the incidence of glioma [16], and the mutation frequency of MSH6 was 7.2% in colorectal cancer and 9.6% in endometrial cancer [17]. Lower PMS2 expression in Lynch syndrome will lead to mismatch repair problems [18], which is a sign of susceptible genes in Lynch syndrome. PMS2 was also reported to be the most frequently mutated Lynch syndrome gene in endometrial cancer [19]. dMMR is the basis of MSI. Studies have found that when DNA slips or repairs during replication, sliding strands mismatch with complementary strand bases, resulting in insertion or deletion of one or several repeating units, the large changes in the number of repetitive copies can lead to functional alterations of certain important genes, which in turn lead to the generation of MSI [20]. MSI has been shown to be the most sensitive and specific indicator of dMMR. The experiment of MSI and MMR in 518 kinds of cancer tissues showed that the consistency of MSI and MMR was 98.3% [21], which is in agreement with the 94% result obtained by E. Stelloo et al. [22].

The occurrence of tumors is a complex biological process involving multiple genes and factors. The MMR system plays a key role in maintaining genome stability, and its mutation manifests as MSI, which in turn leads to the occurrence of tumors. Studies have found that the occurrence of a variety of tumors may be related to the abnormality of the mismatch repair system [23]. Therefore, this pathway has received increasing attention in the study of various solid tumors. At present, there are as yet no reports on how specific MMR genes impact tumor growth in CC cells. However, previous research found that mutations in MMR genes can cause functional defects, thereby increasing the mutation rate of oncogenes or tumor suppressor genes, which can lead to tumor susceptibility. Secondly, with the malignant growth of cancer cells, they will gradually lose their response to TGF-β, dMMR can cause mutations in some functional genes that regulate cell growth, such as TGF-β, causing cell growth out of control until tumors occur. The further mechanism remains to be studied. A correct understanding of the mechanism of MMR, being able to correctly identify insert and delete it during the replication process, can prevent the development of tumors. In recent years, with the increase of CC incidence caused by MSI [24, 25]. It is becoming increasingly important to study its pathogenesis and treatment. Women with MMR gene mutations were found to have an increased risk of CC [26]. The role of the MSI status as a predictive marker remains controversial. Numerous studies established the value of the MSI status as a prognostic factor in all stages of colorectal cancer. This prognostic value has also been found in gastric and thyroid cancers. We speculated whether this prognostic value would also apply in CC. In cervical intraepithelial neoplasia (CIN) and squamous cell carcinoma (SCC) of the cervix, the overall survival of MSI positive patients was significantly worse than that of microsatellite stable patients. In our study, the relationship between MSI and patient prognosis has not yet been found [27–30]. However, there are few studies on the pathogenesis and related regulatory factors of MMR in CC patients at present. Our study aims to analyze the expression of PD-L1 and the related regulatory factors of PD-L1 expression in CC, and to provide a theoretical basis for the clinical treatment of this antibody drug in CC.

In this study, we examined the expression of PD-L1, the MMR status, and the expression situation of MLH1, MSH2, MSH3, and MSH6 proteins in 118 CC patients. We found that the positive rate of PD-L1 was 58. 47%, the detection rate of dMMR status was 28.8%. Meng et al., reported that 64% of CC patients showed PD-L1 expression [31], while another study showed that PD-L1 was expressed in 59.1% of CC patients [32]. Relevant studies have shown that there was a significant correlation between the expression status of MMR proteins and the clinicopathologic characteristics of a variety of tumors [33–35]. We found that CC patients with a history of miscarriage and a history of births ≥ 3 had an increased chance of developing dMMR status in their TCs. Since more deliveries and miscarriage incidence may lead to higher dMMR rates, we hypothesized that these two factors may be significant for women’s health, which is consistent with the findings of Feng et al. [32]. We also further analyzed the expression of PD-L1 in dMMR and pMMR, the results showed that there was a significant difference in the expression of PD-L1 in TCs of CC patients with dMMR and pMMR status. The relationship between PD-L1 and dMMR has been similarly reported [36]. Combined with the effect of PD-L1 antibody monotherapy on various dMMR type solid tumors. It is speculated that the altere dMMR status was closely related to the expression of PD-L1 protein in TCs. It has been reported that the relationship between PD-L1 and dMMR may be related to the increase in neoantigen load caused by dMMR induced immune recognition [37]. An experiment once analyzed high frequency amplified T cell clones in patients’ peripheral blood and the antigens produced by TCs due to mutations, it was found that TCs will produce thousands of mutation loads on average due to the lack of MMR system, thereby generating a large number of tumor antigens, these antigens were considered to be the main targets of anti-tumor T cell immune response enhanced by PD-1 antibody. We analyzed that MSI caused by dMMR will induce gene mutation in TCs, which will lead to the generation of new antigens, these new antigens were easily recognized by the body’s own immune system because of abnormal structure, which will activate the body’s anti-tumor immune response. Meanwhile, due to the existence of negative feedback mechanism, when dMMR activates the anti-tumor immunity of the body, it will increase the expression of immunosuppressive factors at the same time, which will activate the PD-1/PD-L1 immune pathway of T cells and inhibit the activity of T cells, these immunosuppressive factors, especially γ-IFN, will produce a strong stimulation effect on the expression of PD-L1 of the TCs when the content of γ-IFN increases. So it plays an important role in the regulation of the expression of PD-L1 in the TCs [38, 39]. Higher PD-L1 expression in dMMR endometrial tumors supports the clinical efficacy of PD-L1 inhibitors in the treatment of solid tumors, with significantly better objective response rates and disease control rates [40]. Moreover, PD-L1 expression elevated with an increase in TIL abundance within the endometrial tumor microenvironment, suggesting that PD-1 inhibitors could trigger a potent anti-tumor immune response due to the high PD-L1 expression [41, 42]. We also found that the expression of PD-L1 in TCs was closely related to the expression of DNMTs. There are five members in the DNMT family, including DNMT1, DNMT2, DNMT3a, DNMT3b, and DNMT3L, the most common mutation of DNMT in cancer is the DNMT3a mutation, while DNMT3b is a pro-carcinogenic gene in endometrial, lung and prostate cancer, and a tumor suppressor gene (TSG) in lymphoma. DNMT1 is involved in the maintenance of sequence methylation during cell proliferation, which is required for the maintenance of DNA methylation patterns as well as the aberrant silencing of TSGs in TCs, the primary role of DNMT3a and DNMT3b is de novo methylation [43]. Our study found that PD-L1 was moderately correlated with increased DNMT1 and DNMT3a and strongly correlated with decreased DNMT3b expression. This drives us to study the association between DNA methyltransferase expression and PD-L1 levels. Studies have shown that DNMT1 profoundly enhances PD-L1 expression by IFNβ, and IFNβ can directly upregulate PD-L1 expression through IFNAR1-STAT1 signaling. We also discovered the indirect connection between MMR related proteins and DNMTs in the DNA repair process. DNMT1 as a potential component of the MMR pathway, DNMT1’s presence may prevent the loss of DNA methylation during repair, which may lead to epigenetic deregulation and genomic instability. Although the specific regulatory mechanism between DNMTs expression and PD-L1 level is still unclear, our research confirms the above inference to some extent. The specific regulation situation needs further study [44, 45]. There was a significant difference in the expression of PD-L1 in TCs from CC patients with dMMR and pMMR status. To validate this finding, silencing the MLH1 and MSH2 genes in Siha and Caski CC cell lines through RNAi technology to simulate the formation of stable dMMR cell lines, followed by the study of the expression of PD-L1 and DNMTs in the dMMR CC cells. It was found that in Siha cells, under different MMR states, the PD-L1 levels were significantly increased at both the mRNA and protein levels, and the expression levels of DNMT3a and DNMT3b were increased in both MLH1-KD and MSH2-KD groups compared with the NC group. In Caski cells, the highest abundance of PD-L1 gene expression was 1.477 times that of NC group, there was no significant difference in PD-L1 protein expression, and the abundance of DNMT3b protein expression was increased in both MLH1-KD and MSH2-KD groups compared with the NC group. There have been similar experiments, the authors found that the expression levels of cGAS-STING pathway genes in tumor tissues was significantly positively correlated with the prognosis of patients with dMMR tumors, but this phenomenon was not found in pMMR tumors. To verify this phenomenon, the authors knocked down the key MMR gene MLH1 and established a dMMR mouse tumor model to further investigate the relationship between the cGAS-STING pathway and the effect of immunotherapy in patients with dMMR tumors [46]. Found that the expected experimental effect could be achieved by knocking down the key gene.

Then, Siha and Caski CC cells were infected with NC, MLH1-KD, and MSH2-KD knockdown lentiviruses, and the lentivirus-infected Siha and Caski cells were drug screening and cell expansion. Given that the Siha cells used in this experiment were of human origin, immunodeficient nude mice without thymus were chosen for the subcutaneous tumor formation experiments to prevent immune rejection triggered by species differences. It was found that subcutaneous inoculation of Siha cells infected by viruses in NC, MLH1-KD and MSH2-KD groups had a better effect on tumorigenesis in nude mice. Compared with NC group, the tumor weight in MSH2-KD group decreased. By quantitative PCR analysis, we found that in Siha cells the abundance of MLH1 gene expression in the MLH1-KD group was 0. 495 times higher than that of NC group, the abundance of MSH2 gene expression in the MSH2-KD group was 0. 352 times higher than that of NC group, the expression of PD-L1 in the tumor specimens of both groups was strongly positive, and the positivity rate was 100%, further confirming that dMMR system can promote high PD-L1 expression in CC. The study showed that the expression of methyltransferase was lower in the tumor of Siha cells with dMMR status than that of normal Siha cells, while the expression of PD-L1 was relatively higher. Compared to mRNA, proteins are more stable and usually exist at higher magnitudes within cells, reducing the chance of their fluctuations. Therefore, the increase in mRNA levels was significantly higher than that of protein levels. It also shows that abnormal MMR had a very complex mechanism in regulating the increase in PD-L1 expression [47]. Many mechanisms have been shown to regulate its expression, including multiple signaling pathways, transcription factors, and post-transcriptional regulators. This study combined the functions of dMMR and speculated that the main reasons why dMMR affects PD-L1 expression include two aspects. First, up-regulation of protein expression by copy number changes. That is to say, dMMR may destroy the 3’ untranslated region (3’-UTR) of the PD-L1 gene, the destruction of the PD-L1 3’-UTR includes deletion, insertion, translocation, and tandem duplication of the gene sequence, may lead to abnormal amplification of the PD-L1 gene, ultimately leading to abnormally high expression of PD-L1 protein. At the same time, it has also been reported that MSI caused by MMR defects often leads to abnormal tandem repeat of gene sequences. Second, the epigenetic changes related to PD-L1 protein. Epigenetic changes include gene silencing, DNA methylation, nucleolar dominance, activation of dormant transposons, and genomic imprinting. The most important form is methylation modification of promoters in gene coding regions, by demethylating the promoter region of PD-L1 gene, the body can high expression PD-L1, which is one of the main pathways to regulate the expression of PD-L1 [48, 49].

There are some limitations of this study. First, this is a retrospective study, there was a relatively small sample size and potential selection bias. It needs to be verified in the future with a larger sample size. Second, CC cell lines encompass various categories, we only validate Siha and CaSki strains. Third, Caski cells did not induce tumorigenesis in nude mice after virus infection and were excluded from subsequent experiments. The same study needs to be conducted in several other cell lines to verify.

## Conclusions

PD-L1 is universal expressed on the surface of CC cells, dMMR status enhances the expression of PD-L1 on the surface of CC cells. Defects in the MMR system in CC are associated with the demethylation status of the promoter region of the PD-L1 gene.

## Data Availability

The datasets supporting the conclusions of this article are included within the article.

## Abbreviations

CC: Cervical cancer
DNMTs: DNA methyltransferase
MMR: Mismatch repair
TCs: Tumour cells
RNAi: RNA interference
dMMR: MMR deficiency
pMMR: MMR proficient
MSI: Microsatellite instability
IHC: Immunohistochemistry
qRT-PCR: Quantitative real-time PCR
TCGA: The Cancer Genome Atlas
